# A pilot study of assessing whole genome sequencing in newborn screening in unselected children in China

**DOI:** 10.1002/ctm2.843

**Published:** 2022-06-05

**Authors:** Min Jian, Xiaohong Wang, Yuanyuan Sui, Mingyan Fang, Chenchen Feng, Yingping Huang, Chunhua Liu, Ruidong Guo, Yuanning Guan, Yuxiao Gao, Zhiwei Wang, Shuli Li, Bochen Cheng, Lina Sun, Fenghua Cui, Jia Guo, Ying Zhan, Guohong Zhang, Ling Zheng, Fengxia Su, Wei Xue, Puyi Qian, Shaobo Gao, Jiayu Chen, Lingyao Guan, Haorong Lu, Karsten Kristiansen, Xin Jin, Fang Chen, Yuhuan Zhao, Lennart Hammarström, Xiaojing Jiang, Junnian Liu, Ya Gao

**Affiliations:** ^1^ BGI‐Shenzhen Shenzhen China; ^2^ China National GeneBank, BGI‐Shenzhen Shenzhen China; ^3^ BGI‐Qingdao, BGI‐Shenzhen Qingdao China; ^4^ Maternal and Child Health and Family Planning Service Center of Huangdao District Qingdao China; ^5^ Traditional Chinese Medical Hospital of Huangdao District Qingdao China; ^6^ The Affiliated Hospital of Qingdao University Qingdao China; ^7^ Qindgao West Coast New Area Central Hospital Qingdao China; ^8^ Guangdong Provincial Key Laboratory of Genome Read and Write, BGI‐Shenzhen Shenzhen China; ^9^ Department of Biology University of Copenhagen Copenhagen Denmark; ^10^ Medical Career Development Center, Huangdao District Qingdao China; ^11^ Shenzhen Engineering Laboratory for Birth Defects Screening Shenzhen China

Dear Editor,

We investigated whether screening by whole genome sequencing (WGS) in unselected newborns provides more information of potentially curable or treatable medical conditions than routine newborn screening (NBS). We demonstrated that compared with routine NBS, WGS produced fewer false positive results and identified more actionable pathogenic or likely pathogenic variants in the selective 246 genes.

Previously, WGS has been used to identify mutated genes in newborn children with a suspected disease.^1^ However, sequencing of apparently healthy newborns has remained controversial due to technical concerns and ethical issues.^2^ In this study, 321 non‐pre‐selected newborns from a cohort of pregnant women in Qingdao, China were recruited (Table 1). DNA from 303 umbilical cord blood samples and 18 umbilical cords was extracted for 40X WGS. For data interpretation, we selected 251 genes associated with 59 Mendelian disorders, 164 primary immunodeficiency diseases (PIDs) and five pharmacogenetic (PGx) genes, following the guidelines by the Recommended Uniform Screening Panel (RUSP), the International Union of Immunologic Societies (IUIS) Expert Committee for Primary Immunodeficiency, the Dutch Pharmacogenetics Working Group (DPWG), and the Clinical Pharmacogenetics Implementation Consortium (CPIC).^3^, ^4^, ^5^ Sequencing protocol, data analysis pipeline, and criteria for sequence variants interpretation following the ACMG/AMP guidelines are described in the Supporting Information. The WGS results were compared with NBS results, including the mandatory checks of hearing impairment and four metabolic diseases, the metabolic testing of 48 inherited metabolic diseases (IMDs), and the genetic screening for 20 hearing loss loci incorporated into the local NBS program in China.^6^, ^7^

**TABLE 1:** Summary of the demographic data collected from the 321 newborns of Qingdao cohort

	Type	Number	Percentage (%)
Pregnancy	Natural pregnancy	306	95.4
	Assisted reproduction technology	11	3.4
	Unspecified	4	1.20
Gestational weeks	Pre‐term birth	7	2.2
	Term birth	314	97.80
	Average delivery gestation	39 weeks plus 5 days	–
Gender of newborn	Male	151	47.04
	Female	170	52.96
Parental age at delivery	Father's age (ave. year)	33	–
	Mother's age (ave. year)	32	–
	SD of father's age (year)	5	–
	SD of mother's age (year)	4	–
Mandatory NBS screening of 4 metabolic diseases (PKU, CAH, CH and G6PD) by TRFIA	Phe +	1	0.31
	Negative	320	99.69
Mandatory hearing impairment screening by OAEs or AABR	Not passed	0	0.00
	Passed	321	100.00
48 IMDs screening by tandem MS/MS	C5‐OH +	1	0.31
	Phe +	1	0.31
	Negative	310	96.57
	Unspecified	9	2.80
Genetic hearing loss screening by MALDI‐TOF	Carrier	18	5.61
	Negative	294	91.59
	Unspecified	9	2.80

AABR, automated auditory brainstem response; CAH, congenital adrenal hyperplasia; CH, congenital hypothyroidism; CH G6PD, glucose‐6‐phosphate dehydrogenase; C5‐OH+, isovalerylcarnitine positive; MS/MS, mass spectrometry; MALDI‐TOF, matrix‐assisted laser desorption/ionization time of flight; OAEs, otoacoustic emissions; PKU, phenylketonuria; Phe+, phenylalanine positive; SD, standard deviation; TRFIA, time resolved fluoroimmunoassay.

Among the analysed DNA samples of 321 newborns, the average sequencing depth was 47.42X (28.84X–82.90X) and the average coverage was 99.48% (99.01%–99.89%) (Figure 1A). For the 59 Mendelian disorders, a total of 131 pathogenic or likely pathogenic (P/LP) mutations and 5 pathogenic copy number variations were detected in 107 of the 321 newborns (33.33%), corresponding to 106 carriers of 28 diseases and 1 patient with phenylketonuria (PKU) (Figure 1B and Table 2). The 25.23% of newborns (*n* = 81) carried one P/LP mutations, and 7.17% and 0.93% of newborns (*n* = 23 and *n* = 3) carried two or three P/LP mutations, respectively. Hearing loss, methylmalonic acidemia (MMA), primary congenital hypothyroidism (CH), and PKU were diseases with the most carriers, while *GJB2* (28/321, 8.72%), *MMACHC* (11/321, 3.43%), *DUOX2* (10/321, 3.12%), *PAH* (8/321, 2.49%) and *SLC26A4* (8/321, 2.49%) were the top five genes with the highest carrier frequencies of P/LP mutations (Table 2).

FIGURE 1Overview of the results of newborn WGS in the Qingdao cohort (*n* = 321). (A) The sequencing quality of WGS results in the Qingdao cohort (*n* = 321). (B) Overview of carriers and/or patient identified by WGS in the Qingdao cohort (*n* = 321). A total of 107 carriers and/ or patients associated with 34 inherited disorders were identified in the 321 newborns. Of the 136 P/LP mutations, 42.65% corresponded to genetic disorders, 6.62% were associated with PIDs and 50.74% were related to metabolic disorders, including organic acid conditions (14.71%), amino acid disorders (14.71%), fatty acid oxidation disorders (7.35%) and endocrine disorders (13.97%). Hearing loss, MMA, CH and PKU were the most common diseases with carriers, while *GJB2* (28/321, 8.72%), *MMACHC* (11/321, 3.43%), *DUOX2* (10/321, 3.12%), *PAH* (8/321, 2.49%) and *SLC26A4* (8/321, 2.49%) were the top five genes with the highest carrier frequencies of P/LP mutations. For the 164 PIDs recognized by the IUIS, 9 heterozygous P/LP variants in 6 genes, corresponding to 6 diseases, were identified in 9 of the 321 newborn children (2.80%), all in a heterozygous state. Of these, there are two genes (*ADA* and *JAK3*) for which we found two carriers. (C) Distribution of actionable PGx variants identified by WGS in the Qingdao cohort (*n* = 321). A clinical management strategy can be adopted for every carrier according to the DPWG guidelines. Among 321 newborns, 313 newborns (97.51%) in the Qingdao cohort carried at least one clinically relevant variant, while 193 newborns (60.12%) harboured two and/or three variants. From the perspective of the five crucial pharmacogenes, the *CYP2D6* gene had the highest carrier frequency, where 266 out of the 321 infants (82.87%) harboured at least one actionable PGx variant. The gene *CYP2C19* showed the second‐highest carrier rate, where 209 infants (65.11%) carried at least one clinically relevant variant. In addition, 133 and 122 infants carried actionable PGx variants at the gene *UGT1A1* and *NUDT15*, respectively. No actionable variant was identified in the *DPYD* gene in the 321 children. Among the selected gene–drug pairs, irinotecan, azathioprine, mercaptopurine and tioguanine were prescription drugs to paediatric patients, while codeine and clopidogrel are restricted for use of children under 18 years old. Our findings suggest that participants may obtain benefit from PGx profiling already in early childhood

**Figure:**
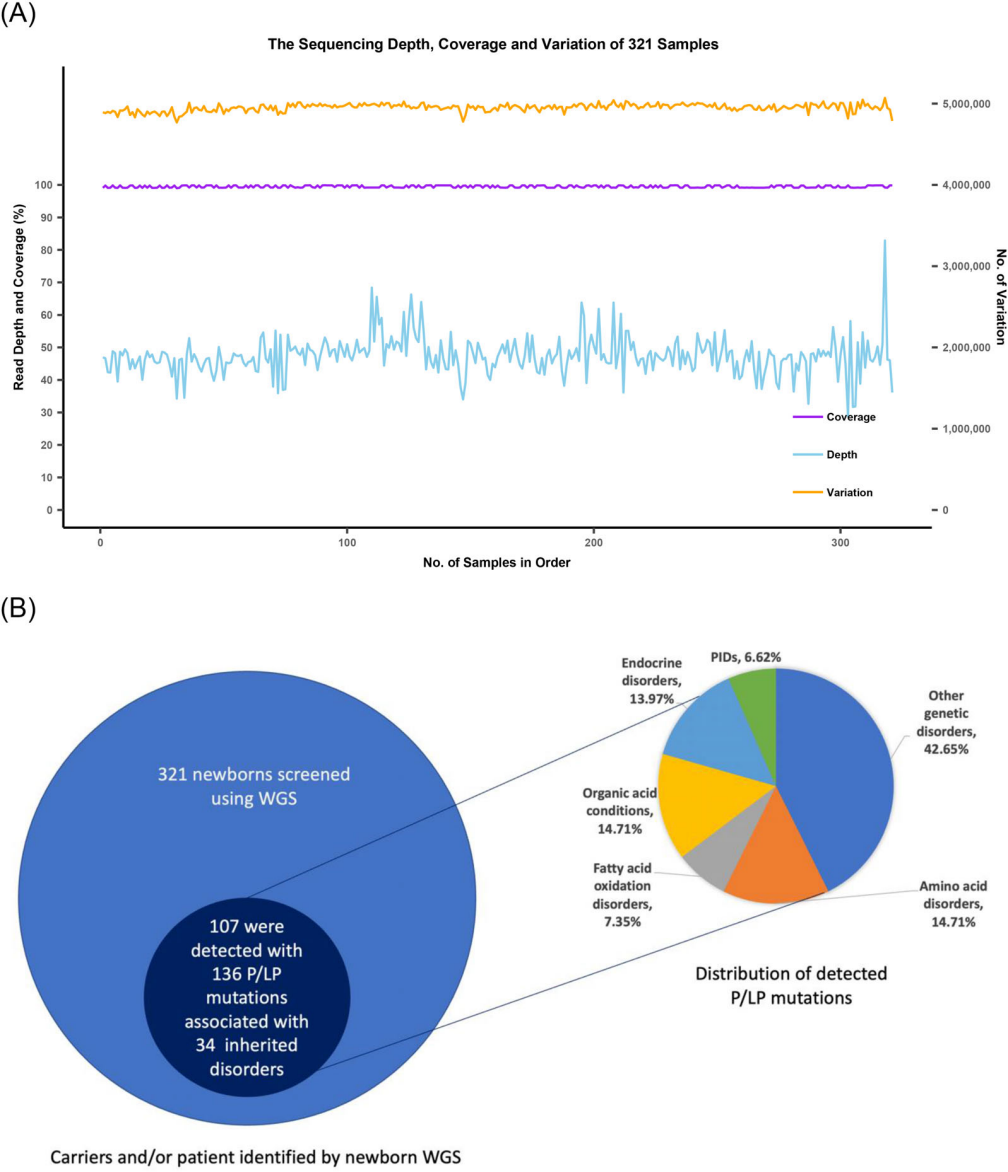

**Figure:**
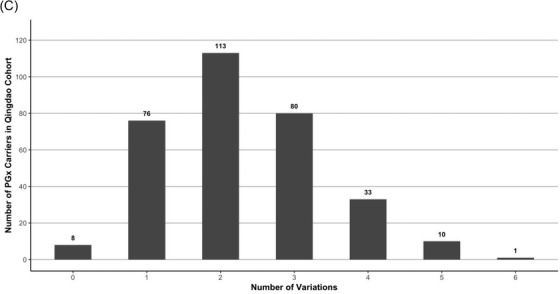

**TABLE 2:** Overview of the P/LP mutations identified in 321 newborn children from Qingdao.

Condition	Inheritance	Gene	Variation	Protein	Classification	Num of newborns	Het/Hom
Propionic acidemia	AR	*PCCB*	c.1364A > G	p.Y455C	P	1	Het
			c.793G > A	p.G265S	LP	1	Het
Methylmalonic acidemia (methylmalonic‐CoA mutase)	AR	*MUT*	c.2179C > T	p.R727*	P	1	Het
Methylmalonic acidemia (cobalamin disorders)	AR	*MMAA*	c.658G > A	p.V220M	LP	1	Het
	AR	*MMACHC*	c.315C > G	p.Y105*	P	1	Het
			c.445_446del	p.C149Hfs32	P	1	Het
			c.482G > A	p.R161Q	P	2	Het
			c.609G > A	p.W203*	P	3	Het
			c.658_660del	p.*220del	P	3	Het
			c.80A > G	p.Q27R	P	1	Het
Methylmalonic acidemia (cobalamin disorders)/methylmalonic acidemia with homocystinuria	AR	*MMADHC*	c.748C > T	p.R250*	P	1	Het
3‐Methylcrotonyl‐CoA carboxylase deficiency	AR	*MCCC1*	c.639+2T > A	/	P	1	Het
Holocarboxylase synthase deficiency	AR	*HLCS*	c.782del	p.G261Vfs20	P	2	Het
Glutaric acidemia type I	AR	*GCDH*	c.1213A > G	p.M405V	P	1	Het
Carnitine uptake defect/carnitine transport defect	AR	*SLC22A5*	c.1472C > G	p.S491C	P	4	Het
			c.468G > A	p.W156*	P	1	Het
Medium‐chain acyl‐CoA Dehydrogenase deficiency	AR	*ACADM*	c.548_551del	p.T183Rfs4	P	2	Het
Trifunctional protein deficiency	AR	*HADHB*	c.1175C > T	p.A392V	LP	1	Het
Citrullinemia, type I	AR	*ASS1*	c.919C > T	p.R307C	P	1	Het
			c.352G > A	p.A118T	LP	1	Het
Classic phenylketonuria	AR	*PAH*	c.1301C > A	p.A434D	LP	1	7Het; 1 individual with compound Het
			c.611A > G	p.Y204C	P	2	
			c.728G > A	p.R243Q	P	4	
			c.740G > T	p.G247V	P	1	
			c.842+2T > A	/	P	1	
Primary congenital hypothyroidism	AR,AD	*TSHR*	c.1349G > A	p.R450H	P	4	Het
	AR	*DUOX2*	c.1588A > T	p.K530*	P	6	Het
			c.1883del	p.K628Rfs11	P	1	Het
			c.1946C > A	p.A649E	LP	1	Het
			c.605_621del	p.Q202Rfs93	P	1	Het
			c.3329G > A	p.R1110Q	P	1	Het
	AR	*TPO*	c.2422del	p.C808Afs24	P	1	Het
Congenital adrenal hyperplasia	AR	*CYP21A2*	c.518T > A	p.I173N	P	2	Het
			c.92C > T	p.P31L	P	2	Het
S,S disease (sickle cell anemia)/S, beta‐thalassemia/S,C disease/other haemoglobinopathies	AR	*HBB*	c.126_129del	p.F42Lfs19	P	1	Het
Cystic fibrosis	AR	*CFTR*	c.2052_2053insA	p.Q685Tfs4	P	1	Het
Classic galactosemia	AR	*GALT*	c.821‐7A > G	/	P	1	Het
	AR	*GALT*	c.844C > G	p.L282V	LP	1	Het
Glycogen storage disease type II (Pompe)	AR	*GAA*	c.2237G > C	p.W746S	P	1	Het
			c.2662G > T	p.E888*	P	1	Het
			c.2647‐7G > A	/	LP	1	Het
Hearing loss	AD, AR, DD (digenic dominant)	*GJB2*	c.109G > A	p.V37I	P	14	26Het; 2 individuals with compound Het
			c.235del	p.L79Cfs3	P	10	
			c.299_300del	p.H100Rfs14	P	4	
			c.605_606insAGAAGACTGTCTTCACAGTGTTCATGATTGCAGTGTCTGGAATTTG	p.C202*	P	2	
	AR	*SLC26A4*	c.1174A > T	p.N392Y	P	1	Het
	AR		c.1229C > T	p.T410M	P	1	Het
	AR		c.1262A > C	p.Q421P	LP	1	Het
	AR		c.2027T > A	p.L676Q	LP	2	Het
	AR		c.2168A > G	p.H723R	P	1	Het
	AR		c.919‐2A > G	/	P	2	Het
	AR	*USH2A*	c.2802T > G	p.C934W	P	1	Het
			c.100C > T	p.R34*	P	1	Het
			c.8559‐2A > G	/	P	1	Het
	Maternal	*MT‐RNR1*	m.1095T > C	/	P	4	Hom
Spinal muscular atrophy	AR	*SMN1*	c.(723+1_724‐1)_(834+1_835‐1)del	p.I242_M278del	P	4	Het
			c.(723+1_724‐1)_(885+1_886‐1)del	p.I242_L294del	P	1	Het
Short‐chain acyl‐CoA dehydrogenase deficiency	AR	*ACADS*	c.1031A > G	p.E344G	P	1	Het
Glutaric acidemia type II	AR	*ETFDH*	c.1211T > C	p.M404T	LP	1	Het
Citrullinemia, type II	AR	*SLC25A13*	c.1180+1G > A	/	P	1	Het
			c.852_855del	p.M285Pfs2	P	2	Het
Hypermethioninemia	AR	*GNMT*	c.149T > C	p.L50P	LP	1	Het
Biopterin defect in cofactor biosynthesis/biopterin defect in cofactor regeneration	AR	*PTS*	c.166G > A	p.V56M	P	1	Het
			c.259C > T	p.P87S	P	1	Het
			c.84‐291A > G	/	P	3	Het
Galactoepimerase deficiency	AR	*GALE*	c.505C > T	p.R169W	P	1	Het
Adenosine deaminase (ADA) deficiency	AR	*ADA*	c.424C > T	p.R142*	P	1	Het
			c.872C > T	p.S291L	P	1	Het
Ataxia‐telangiectasia	AR	*ATM*	c.67C > T	p.R23*	P	1	Het
Immunoskeletal dysplasia with neurodevelopmental abnormalities (EXTL3 deficiency)	AR	*EXTL3*	c.1970A > G	p.N657S	P	1	Het
JAK3 deficiency	AR	*JAK3*	c.1744C > T	p.R582W	LP	1	Het
			c.307C > T	p.R103C	LP	1	Het
DNA ligase IV deficiency	AR	*LIG4*	c.1271_1275del	p.K424Rfs20	P	1	Het
TACI deficiency (immunodeficiency, common variable)	AD/AR	*TNFRSF13B*	c.542C > A	p.A181E	LP	2	Het

Het, heterozygous; Hom, homozgyous. P, pathogenic; LP, likely pathogenic.

For the 164 PIDs, 9 heterozygous P/LP variants in 6 genes were identified in 9 newborns (2.80%), all in a heterozygous state (Table 2). Four newborns were shown to carry heterozygous variant of unknown significance (VUS) in the gene *SLC25A13* (c.2T>C, p.M1T), which was predicted as start loss and likely affecting the initiator methionine of the SLC25A13 mRNA. Two newborns carried a VUS in *ASS1*(c.‐4C>T, p.?). Although these VUSs were not included in the final report to the participants, follow‐up of the children with VUSs will be conducted till 3 years of age.

Sanger sequencing confirmed 143 out of 145 mutations identified by WGS, resulting in an accuracy of 98.62%. Carriers of *SMN1* mutations were validated by multiplex ligation‐dependent probe amplification and real‐time quantitative PCR, showing that five out of the six predicted carriers were true.

Of the 321 newborns, 312 (97.20%) had the results of 48 IMDs screening and genetic hearing loss screening on 20 loci, which identified one newborn with PKU and one infant with increased blood level of isovalerylcarnitine (Table 1). In addition, 18 carriers harbouring 20 pathogenic mutations causing hearing impairment were detected by genetic hearting loss screening, albeit all 321 children passed the physical hearing screening at hospital (Supporting Information). The newborn WGS also identified the PKU case and 18 hearing loss carriers (Figure 2A). However, the child with increased level of isovalerylcarnitine was confirmed to be a carrier of 3‐methylcrotonyl‐CoA carboxylase deficiency by WGS. In addition, WGS identified two infants carrying compound heterozygous P/LP variants in *GJB2* (Figure 3) and four children carrying pathogenic mutations in *MT‐RNR1 (c1095T >* *C)*, suggesting an increased risk of late‐onset deafness or drug‐induced hearing loss, respectively. Although currently non‐symptomatic, the two newborns with *GJB2* variants were scheduled to undergo hearing tests every 6 months, and the four newborns with the m.1095T mutation in *MT‐RNR1* were advised to avoid using aminoglycosides.

**FIGURE 2:**
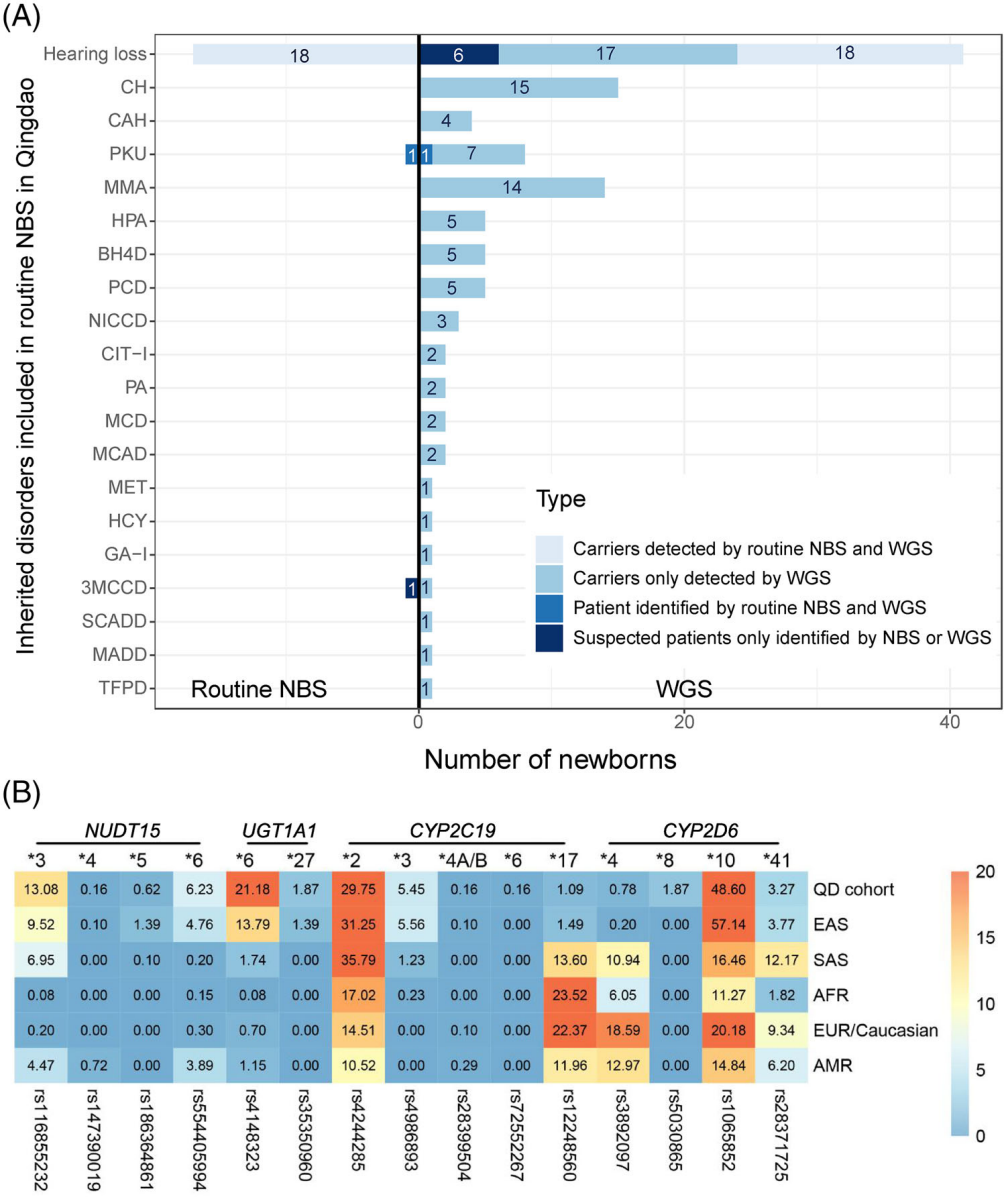
(A) Comparison between the findings of newborn WGS and routine NBS in the Qingdao cohort (*n* = 321). The findings of routine NBS tests are summarized on the left side of (A). In total, 18 carriers of hearing loss associated genes, one patient with PKU and one false positive result of C5‐OH were detected by existing routine methods. The findings of WGS are shown on the right side of (A). The WGS results confirmed the positive routine NBS findings of 18 carriers of hearing loss and the case with increased level of Phe. However, the infant with a routine NBS showing an increased level of C5‐OH (sample ID 18110806) was found to carry one pathogenic mutation in *MCCC1*, corresponding to being a carrier of 3‐methylcrotonyl‐CoA carboxylase deficiency (3MCCD). The newborn WGS also identified more infants carrying extra hearing loss mutations that were not identified by the routine NBS method, including 2 newborns carrying compound heterozygous P/LP variants in *GJB2*, 4 newborns harbouring a pathogenic mutation in *MT‐RNR* and 17 additional carriers harboured altogether 19 variants. Moreover, newborn WGS identified 59 extra carriers carrying 66 P/LP variants corresponding to 18 inherited metabolic diseases that could not be identified by the routine NBS tests. The abbreviations of diseases and the summary of are listed in Table S1. (B) Comparison of allele frequency of actionable PGx variants between the Qingdao cohort and five subpopulations of the 1000 Genome Project dataset, including East Asians (EAS), South Asians (SAS), Africans (AFR), Europeans (EUR) and Americans (AMR). In most cases, the allele frequency of the Qingdao cohort is consistent with the EAS, but differed significantly with the SAS, the AFR, the EUR and the AMR, such as CYP2C19*2, CYP2C19*3 and NUDT15*6. It should be noted, however, that three common PGx variants in the Qingdao cohort, CYP2D6*10 (48.60%), NUDT15*3 (13.08%) and UGT1A1*6 (21.18%), showed significant frequency differences with the EAS (*p* < 0.05), indicating population diversity within the East Asians. Notably, two rare variants, CYP2D6*8 (1.87%, *n* = 11) and CYP2C19*6 (0.16%, *n* = 1) which have not been reported in any subpopulation in the 1000 Genome phase 3 dataset and were first detected in our Qingdao 321 newborns

**FIGURE 3:**
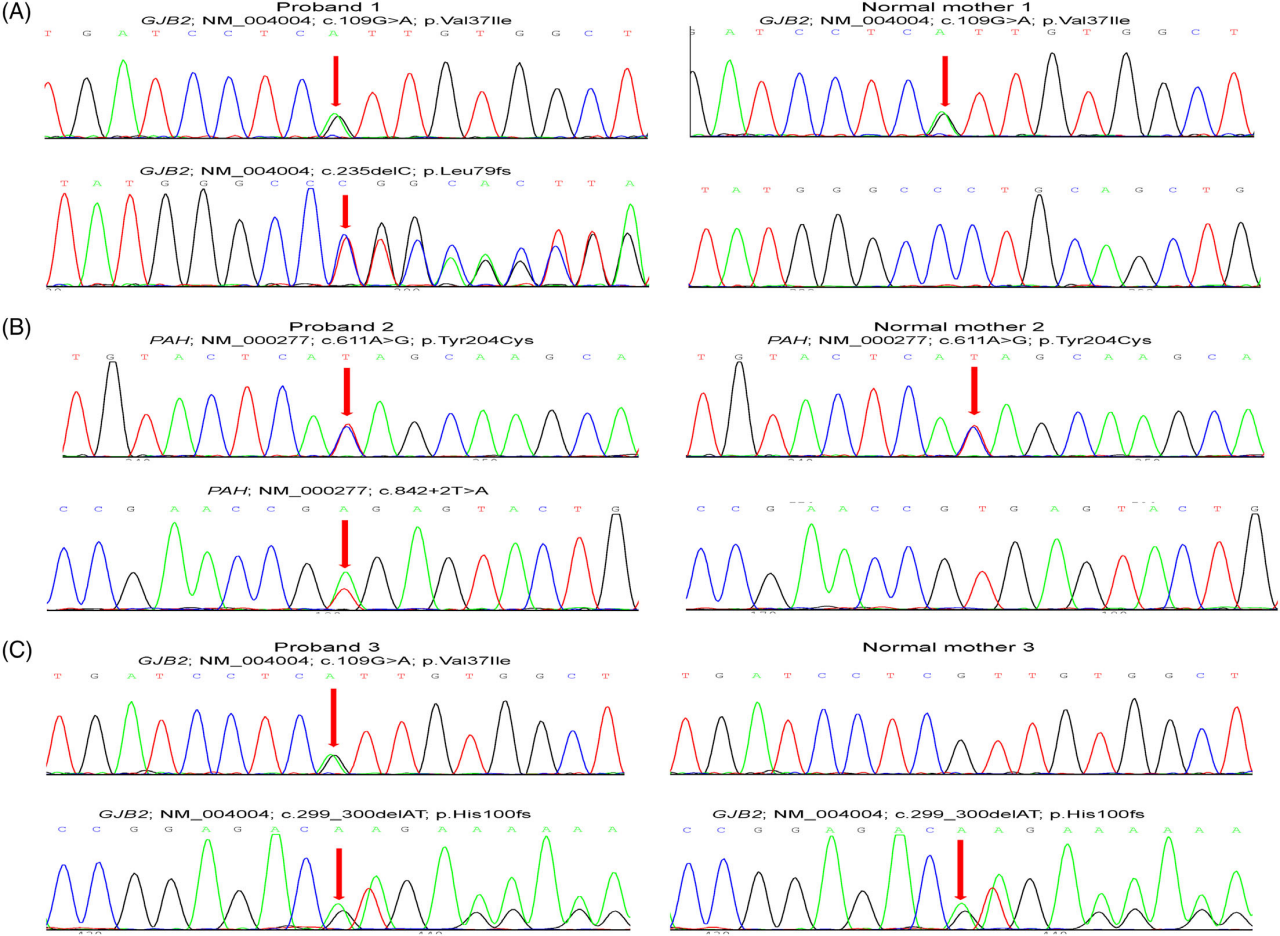
The results of Sanger sequencing and pedigree analysis of three children with compound heterozygous mutations. Three children with compound heterozygous variants were detected by newborn WGS. Two of them had variants at the *GJB2* gen*e* (A) (NM_004004.5, c.109G > A, p.V37I; NM_004004.5, c.235del, p.L79Cfs3); (C) (NM_004004.5, c.109G > A, p.V37I; NM_004004.5, c.299_300del, p.H100Rfs14), and one carried two variants at the *PAH* gene (B) (NM_000277.1, c.611A > G, p.Y204C and NM_000277.1, c.842 + 2T > A). Sanger sequencing confirmed that one variant was inherited from her/his mother. However, as infant father's sample was not available, we could not determine if the small deletion and insertion was inherited from the father or whether it was a de novo variant

Interestingly, we observed that 313 newborns (97.51%) carried at least one actionable PGx variant (Figure 1C). This result is in line with a European 44 000 biobank participants study, where 99.8% of the participants had a genotype associated with increased risks to at least one medication.^8^ Furthermore, we found three common PGx variants in the Qingdao cohort, CYP2D6*10 (48.60%), NUDT15*3 (13.08%) and UGT1A1*6 (21.18%) (Figure 2B and Table 3), that showed significant frequency differences as compared to East Asian populations (*p* < 0.05). An important aspect when screening for disorders in a given population is the use of a matched control database as variants can be highly specific for a given ethnic group.^9^ Most databases published to date are based on individuals of European descent and many populations have limited or poor representation.

**TABLE 3:** Overview of the actionable PGx variants detected in 321 newborns from Qingdao

Gene–drug pairs				Number of carriers in the Qingdao cohort				MAF$					
Drug	Gene	Star allele	dbSNP RS ID	Het	Hom	Variant No.	Carrier No.	Qingdao cohort (%)	EAS (%)	SAS (%)	AFR (%)	EUR/Caucasian (%)	AMR (%)
Azathioprine, mercaptopurine, tioguanine	*NUDT15*	*3	rs116855232	74	5	79	122	13.08	9.52	6.95	0.08	0.20	4.47
		*4	rs147390019	1	0	1		0.16	0.10	0.00	0.00	0.00	0.72
		*5	rs186364861	4	0	4		0.62	1.39	0.10	0.00	0.00	0.00
		*6	rs554405994	36	2	38		6.23	4.76	0.20	0.15	0.30	3.89
Irinotecan	*UGT1A1*	*6	rs4148323	106	15	121	133	21.18	13.79	1.74	0.08	0.70	1.15
		*27	rs35350960	12	0	12		1.87	1.39	0.00	0.00	0.00	0.00
Clopidogrel	*CYP2C19*	*2	rs4244285	141	25	166	209	29.75	31.25	35.79	17.02	14.51	10.52
		*3	rs4986893	33	1	34		5.45	5.56	1.23	0.23	0.00	0.00
		*4A/B	rs28399504	1	0	1		0.16	0.10	0.00	0.00	0.10	0.29
		*6	rs72552267	1	0	1		0.16	0.00	0.00	0.00	0.00	0.00
		*17	rs12248560	7	0	7		1.09	1.49	13.60	23.52	22.37	11.96
Codeine	*CYP2D6*	*4	rs3892097	5	0	5	266	0.78	0.20	10.94	6.05	18.59	12.97
		*8	rs5030865	10	1	11		1.87	0.00	0.00	0.00	0.00	0.00
		*10	rs1065852	150	81	231		48.60	57.14	16.46	11.27	20.18	14.84
		*41	rs28371725	17	2	19		3.27	3.77	12.17	1.82	9.34	6.20

* 1 denotes the default reference (wild type or fully functional) allele or haplotype, while other designations (e.g. *2 or *3) define haplotypes carrying one or more variants.

AFR, africans; AMR, Americans; EAS, East Asians; EUR, Europeans; Het, heterozygous; Hom, homozygous; MAF, minor allelic frequency; SAS, South Asians; $, The MAF of EAS, SAS, AFR, EUR, and AMR refers to the 1000 Genome phase 3 dataset.

Limitations of the current study are the small sample size and restricted metabolic tests. A large‐scale NBS effort is needed to validate our findings and fully investigate the treatable or curable medical conditions in newborns. The technical challenge of newborn WGS is to screen genes with high homology due to the misalignment of short‐read sequencing. Therefore, the customized pipeline is needed to improve the accuracy and sensitivity of SNVs at genes with high‐level homology. Albeit the present cost and turnaround time of WGS is several times more than the present NBS methods, in the forseeable future the pitfalls of WGS cost and turnaround time will likely facilitate the application of newborn WGS in NBS programs.

In our study, selective identification of genomic data, where therapeutic options are available, did not violate the Wilson–Jungner criteria^10^. Our work provides a basis for future research on expanding screening genes and diseases in newborn screening program. Given adequate cost‐effectiveness, WGS should be considered in future newborn screening programs. Further discussion of the interpretation accuracy and ethical use of genomic information needs to take place on a global scale.

## CONFLICT OF INTEREST

The authors declare no conflict of interest.

## Supporting information

Supporting Information

Supporting Information
