# Supplementary material for: A pilot study of assessing whole genome sequencing in newborn screening in unselected children in China
Source: Clin Transl Med. 2022 Jun 5;12(6):e843. doi: 10.1002/ctm2.843 (PMC9167628; doi:10.1002/ctm2.843)
Supplement: Supplementary file 1 — Supporting Information [file CTM2-12-e843-s001.docx]

Contents of Supportive Information

[Supplementaty Background 2](#_Toc100221277)

[Supplementary Methods 3](#_Toc100221278)

[*Study Subjects* 3](#_Toc100221279)

[*Processing of samples* 4](#_Toc100221280)

[*Sequencing* 4](#_Toc100221281)

[*Quality control (QC)* 5](#_Toc100221282)

[*Gene lists* 5](#_Toc100221283)

[*Data Analysis* 5](#_Toc100221284)

[*Variant classification and validation* 6](#_Toc100221285)

[*Reporting and follow-up* 7](#_Toc100221286)

[Supplementary Results 7](#_Toc100221287)

[*Subjects overview* 7](#_Toc100221288)

[*Quality control* 8](#_Toc100221289)

[*Results comparison between routine NBS and newborn WGS* 8](#_Toc100221290)

[*The results of inherited diseases and PIDs* 9](#_Toc100221291)

[*Actionable PGx profiling* 11](#_Toc100221292)

[Supplementary Discussion 12](#_Toc100221293)

[Reference 17](#_Toc100221294)

# Supplementaty Background

Newborn screening for metabolic diseases was initiated in the 1960-ties and was based on the early identification of children with phenylketonuria (PKU) (Føllings disease) as a cause of childhood mental retardation ^1^. The development of an assay based on examination of a dried blood spot (DBS) on a filter paper in 1963 (Guthrie card) ^2^ formed the basis of a technically simple screening assay, allowing the identification and subsequent dietary treatment of affected children.

Extended screening for a large number of additional metabolic diseases, aided by the introduction of mass spectrometry methods, has been gradually implemented, albeit to a different extent in various countries ([https://membership.isns-neoscreening.org/disorders/](https://eur01.safelinks.protection.outlook.com/?url=https%3A%2F%2Fmembership.isns-neoscreening.org%2Fdisorders%2F&data=02%7C01%7Clennart.hammarstrom%40ki.se%7Cb2325ba329344315a7d008d74d0ffac7%7Cbff7eef1cf4b4f32be3da1dda043c05d%7C0%7C0%7C637062603357735613&sdata=s%2FjqTQwuEaSZ55CCkE7%2B2yZ0JN4LUCj1y5SZaQvGWf4%3D&reserved=0)). The ethical guidelines published by Wilson and Jungner in 1968 ^3^, remains the gold standard for which diseases to screen for and the current list of recommended disorders in the US contains 35 core and 26 secondary conditions (Recommended Uniform Screening Panel by the Secretary of the Department of Health and Human Services, RUSP/HHS) (<https://www.hrsa.gov/advisory-committees/heritable-disorders/rusp/index.html>).

DNA based screening using the DBS has recently been introduced, and screening for cystic fibrosis is included in screening programs in selected countries. Severe Combined Immunodeficiency (SCID) (T cell lymphopenia) using quantification of T cell receptor excision circles (TREC) ^4^ has also been implemented, starting in the USA in 2010 ^5^. Screening for kappa receptor excision circles (KREC) (B cell lymphopenia in X-linked agammaglobulinemia (XLA)) followed in 2011 ^6^, and a combined assay is used in selected countries. Currently, testing for other diseases such as Spinal Muscular Atrophy (SMA) is being implemented and additional conditions are being considered for national genetic screening of newborns.

The NSIGHT initiative (NIH) 2012-2018 (https://www.genome.gov/Funded-Programs-Projects/Newborn-Sequencing-in-Genomic-Medicine-and-Public-Health-NSIGHT), aimed at large scale Newborn Sequencing in Genomic Medicine and Public Health using exome sequencing (ES), or whole genome sequencing (WGS). The supported projects were related both to data collection, identification of specific disorders and ethical, legal and social implications of genomic sequencing of newborns.

Different methods for identification of mutated genes, including Targeted Region Sequencing (TRS), ES and WGS, is currently standard procedure in newborn children with a suspected disease. However, sequencing of apparently healthy newborns remains controversial as it may violate the Wilson and Jungner criteria. Yet, ES and WGS has already been performed on small cohorts of “healthy” newborn children ^7,8^. The latter study identified actionable adult onset disease in 3.5% and actionable childhood onset disease in 9.4% of the tested children. Furthermore, relevant pharmacogenomic variants were identified in 5% of the newborns, supporting the concept of introducing ES/WGS based screening of all newborns.

An estimated of 10 million infants are born in China each year, of which 4% - 6% ^9^ are affected by birth defects. Metabolic tests and tandem mass spectrometry (MS/MS) for PKU ^10^, primary congenital hypothyroidism ^10^ , congenital adrenal hyperplasia^11^ as well as limited genetic screening for hearing loss ^12^ are included in the national-wide and/or regional newborn screening programs in China. However, many newborn patients and/or carriers remain genetically undetermined, partly due to the limited gene-by-gene screening strategy. The current study is the largest study to date using WGS on unselected newborns, looking at a wide panel of inherited diseases, primary immunodeficiency diseases and pharmacogenomically relevant variants in China.

# Supplementary Methods

## ***Study Subjects***

This pilot study consecutively enrolled 321 pregnant women at three collaborating hospitals from the Huangdao District, Qingdao, China from May 2018 to December 2018. The involved pregnant women were prospectively recruited while they had their regular visits for routine prenatal screening of chromosome aneuploidy at 13 to 20 gestational weeks. Each pregnant women was given a detailed explanation of the study by professional clinicians and signed informed consent at the recruitment site. For each pregnant woman, umbilical cord blood and/or umbilical cord of each pregnancy were collected at birth for the WGS program in this study. Meanwhile, routine NBS clinical services was still provided to each newborn, including the testing for genetic hearing loss of 20 loci by matrix-assisted laser desorption/ ionization time of flight (MALDI-TOF) mass spectrometry ^12^, 48 metabolic diseases by tandem mass spectrometry (MS/MS) (Supplementary Table 1) and four mandatory NBS screening for phenylketonuria (PKU), primary congenital hypothyroidism, primary congenital hypothyroidism and glucose-6-phosphate dehydrogenase deficiency by time resolved fluoroimmunoassay. Newborn hearing screening at hospital included otoacoustic emissions and auditory brainstem response tests. Newborn WGS results with actionable findings were returned to the family, together with genetic counselling provided by professional clinicians.

The study was approved by the medical ethics committee (Maternal and child health and family planning service center of Huangdao district Ethics Reviews board) and the Institutional Review Board of BGI. Each pregnant woman and her spouse signed an informed consent form before participating in the study.

## ***Processing of samples***

Umbilical cord blood (5 ml) and/or umbilical cord (3 tubes, 1 cm per tube) were collected from each pregnant woman at delivery. When WGS was carried out, umbilical blood DNA was preferred to umbilical cord DNA. Umbilical blood DNA was extracted with the HiPure Blood DNA Mini Kit (Magen, Guangzhou, China) whereas umbilical cord DNA was extracted with a Salting-out Self-dispensing Kit (Magen, Guangzhou, China). After DNA extraction, Qubit 3.0 fluorometer (Life Technologies, Paisley, UK) was used to measure the DNA concentration, and a 2% agarose gel electrophoresis was used to detect DNA fragment integrity.

## ***Sequencing***

Extracted DNA subsequently underwent library construction and was sequenced using the sequencing platform DIPSEQ (MGI, Shenzhen, China) with 100-bp paired-end reads. Briefly, genomic DNA was normalized and processed for circularization ^13^. Genomic DNA was heat-denatured at 95 °C for 3 minutes to make a single strand DNA circle (ssDNA circle), which were then mixed with reagents of the MGIEasyTM DNA Library Prep Kit (MGI, Shenzhen, China) and incubated at 37 °C for 30 minutes to complete the circularization. The resulting ssDNA circles were then used to generate DNA nanoballs (DNBs) by rolling circle amplification (RCA) ^14^. After RCA and the formation of DNBs, the final product was measured by Qubit using the ssDNA HS Assay kit (Invitrogen), and loaded on a DIPSEQ platform (MGI, Shenzhen, China) for sequencing ^15^ following the manufacturer’s instructions.

## ***Quality control (QC)***

In order to ensure high-quality of the data for each sample, stringent quality control criteria were applied, which required the GC content of the sequencing read to fall within 40% - 44%, the average Q30 above 80%, the duplicated rate below 10%, the average depth above 20x, the percentage ≥ 4x coverage of NoN-regions above 96%, the Ti/Tv ratio within 1.96 - 2.02 and the het/hom ratio within 1.3 - 1.7.

## ***Gene lists***

A total of 251 genes associated with 59 inherited metabolic diseases, 164 severe combined immunodeficiency diseases and adverse drug reactions were analyzed (Supplementary Table 2-4). The causative genes for inherited diseases and severe immunodeficiency diseases were selected following the Recommended Uniform Screening Panel and the International Union of Immunologic Societies Expert Committee for Primary Immunodeficiency (IUIS)^16,17^ list of genes.The clinically actionable genes were based on the Dutch Pharmacogenetics Working Group recommendation and Clinical Pharmacogenetics Implementation Consortium guideline (https://cpicpgx.org). Each gene and its inheritance was manually reviewed according to Online Mendelian Inheritance in Man (OMIM) (<https://omim.org>) and the published literature.

## ***Data Analysis***

Burrows-Wheeler Aligner (BWA-0.7.12) was used to align paired-end reads to the National Center for Biotechnology Information (NCBI) reference (GRCh38 / UCSC hg38) and the alternative contigs in GRCh38 assembly were deleted to improve the alignment accuracy. The Genome Analysis Software Kit (GATK 4.0) best practice pipeline was used to perform variants calling including SNVs (Single nucleotide variants) and short InDels (insertions/deletions). The SMN copy-number caller (v1.1), a customized CNV tool, was applied to identify pathogenic SNVs on SMA from the WGS data ^18^. After variation calling, bcftools (v1.9) was used to extract variation from selected genes. For each transcript, all the regions including UTRs, intronic and exon regions were included. If one gene has multiple transcript possibilities, the longest transcript among each of those genes was used. Subsequently, all the samples were merged using bcftools. Polymorphic sites were then annotated by BGI in-house databases and public databases, such as the 1000 Genomes Project (KG, http://www.1000genomes.org/), the Exome Variant Server (ESP, http://evs.gs.washington.edu/EVS/), the Exome Aggregation Consortium (ExAC, http://exac.broadinstitute.org) and the Genome Aggregation Database (gnomAD, https://gnomad.broadinstitute.org).

## ***Variant classification and validation***

The criteria of standards and guidelines for the interpretation of sequence variants by ACMG/AMP published in 2015 were followed for variants classification^19^. Candidate variants that met one of the following preliminary criteria were selected for manual reviewing: (1) Variants classified as pathogenic (P) or likely pathogenic (LP) mutations in the ClinVar database; (2) Variants classified as disease-causing mutations (DM) or probable disease-causing mutations (DM?) in the Human Gene Mutation Database (HGMD); (3) Variants annotated as nonsense, frameshift, stop-gained, stop-lost, initiation codon and splice donor/acceptor; (4) Variants with minor allelic frequency (MAF) < 0.05. Candidate variants were further reviewed by analysts based on typical types of evidence codes of the ACMG/AMP guidelines.

All the variants classified as pathogenic or likely pathogenic (P/LP) and uncertain significance (VUS) favor pathogenic were further validated by Sanger sequencing, as well as actionable PGx variants. If compound heterozygous variants were identified in one infant by WGS, the sample of his/her mother would also be validated by Sanger sequencing. Multiplex ligation-dependent probe amplification (MLPA) and real-time quantitative PCR (qPCR) were applied to confirm the predicted carriers of spinal muscular atrophy (SMA) with one copy loss of the gene *SMN1*.

## ***Reporting and follow-up***

Once the newborn WGS program was completed, the legal guardian of each newborn was provided with a final report, including the results of the genetic testing and interpretation and genetic counseling advice. The turnaround time of newborn WGS in our study ranged from 16 weeks to 24 weeks, from sample collection to report delivery, due to the research base of study.

Only P/LP results on inherited disorders and PGx profiling were returned in the genetic report to participants, while the VUS variants in genes related to the existing phenotype and genes with evidence of causing the specific indication, were documented and returned to research team and collaborated hospitals. This allowed for studies such as segregation analysis or further clinical evaluation that could help clarify their clinical significance. Positive results on inherited disorders were counseled by professional clinicians at collaborative hospitals. In addition, the routine NBS results of genetic hearing loss of 20 loci and 48 metabolic diseases were collected from collaborative hospitals in order to assess the consistency of newborn WGS results in a blinded manner.

This pilot study is a part of a maternal and newborn cohort, which is designed to conducted routine follow-up study for all newborns until three years of age. The follow-up of children with P/LP and VUS variants was coordinated as a part of the routine follow-ups.

# Supplementary Results

## ***Subjects overview***

The newborn WGS was conducted with the DNA from 303 umbilical cord blood samples and 18 umbilical cords. The 321 newborns consisted of 151 (47%) males and 170 (53%) females. Routine NBS records of newborns were recalled during the period of follow-up and the recall rate were 97.20%. The demographic data of 321 newborns are summarized in Table 1.

## ***Quality control***

The average sequencing depth of the 321 samples was 47.42X (28.84X - 82.90X) on a genome-wide scale, and the average coverage was 99.48% (99.01% - 99.89%) (Fig.1A). Among the 251 genes analyzed, 245 genes had over 85% breadth of coverage above 10X. Six genes were flagged with poor coverage (67.35% - 14.71%), mainly due to high homology or a pseudogene causing misalignment. After extensively literature searching, gene *SMN1* copy number caller was applied to analyze carrier/patient of SMA with a reported recall of 100% for SMA and 97.8% for carriers, and a precision of 100% for both SMA and carriers. The other five genes flagged with poor coverage were removed from the gene list, including *IKBKG, CFC1, HBA2, CBS* and *ICOSLG*. After QC, 246 genes were retained for further analysis. The coverage of examined genes of the 321 samples was listed in Supplementary Table 5.

## ***Results comparison between routine NBS and newborn WGS***

Of the 321 newborns, 312 (97.20%) had routine NBS results of hearing loss on 20 loci and 48 metabolic diseases. Among the recalled newborns, routine NBS service identified one newborn with Phe positive result associated with PKU and one infant with an increased level of C5OH corresponding to the a high risk of amino acid diseases. In addition, 18 carriers harboring 20 pathogenic mutations of hearing loss were detected by routine NBS, albeit all 321 children passed the physical hearing check at their hospital. No other diseases were identified by the routine NBS service.

The newborn WGS results confirmed the positive routine NBS findings of 18 carriers of hearing loss and the case with increased level of Phe (Fig.2A and Supplementary Table 6). The Phe case (sample ID 18120159) was identified carrying compound heterozygous mutations in the *PAH* gene (Fig.3B). However, the infant with the routine NBS result of an increased level of C5OH (sample ID 18110806) was found to carry one pathogenic mutation in *MCCC1* by newborn WGS, corresponding to being a carrier of 3-Methylcrotonyl-CoA Carboxylase Deficiency (Fig.2A).

The newborn WGS also identified more infants carrying extra hearing loss mutations that were not identified by the routine NBS method, including 2 newborns (sample ID 18110835 and 18121240) carrying compound heterozygous P/LP variants in *GJB2* (Fig.3A, 3C), 4 newborns (sample ID 18091180, 18091700, 18110851 and 18121234) harboring a pathogenic mutation in *MT-RNR* and 17 additional carriers harbored altogether 19 variants (Fig.2A and Supplementary Table 6). Moreover, newborn WGS identified 59 extra carriers carrying 66 P/LP variants corresponding to 18 inherited metabolic diseases that could not be identified by the routine NBS tests (Fig.2A and Supplementary Table 6). Sanger sequencing confirmed the findings of the newborn WGS.

Follow-up of further clinical diagnosis confirmed classic PKU of the child with compound heterozygosity in *PAH*, while the infant with an increased level of C5OH was confirmed to be false positive of routine NBS. Of note, the six children with mutations in *GJB2* and *MT-RNR1* have had no signs of hearing loss yet. After genetic counseling, the two newborns with *GJB2* variants were scheduled to undergo hearing testing every six months, and the four newborns with the m.1095T mutation in *MT-RNR1* were adviced to avoid using aminoglycosides.

## ***The results of inherited diseases and PIDs***

In the present study, newborn WGS was performed with >30X sequencing depth with an average coverage of 99.48%. As a result, the newborn WGS identified 136 P/LP mutations associated with 32 inherited disorders from 107 infants (33.33% of samples). Of these mutations, 42.65% were corresponding to the other genetic disorders, 6.62% were associated with primary immunodeficiency disorders, and 50.74% were related to metabolic disorders, including organic acid conditions (14.71%), amino acid disorders (14.71%), fatty acid oxidation disorders (7.35%) and endocrine disorders (13.97%) (Fig.1B).

For 59 inherited diseases included in the Recommended Uniform Screening Panel, a total of 127 P/LP mutations and five pathogenic copy number variations (CNV) were detected in 102 of the 321 newborns (31.78%), corresponding to 101 carriers of 26 diseases and 1 patient with PKU (Table 2 and Supplementary Table 6). In general, 25.23% of newborns (81) carried one P/LP mutations, and 7.17% and 0.93% of newborns (23 and 3) carried two or three P/LP mutations (Supplementary Table 7), respectively. Hearing loss, methylmalonic acidemia, congenital hypothyroidism and PKU were the most common diseases with carriers, while *GJB2* (28/321, 8.72%), *MMACHC* (11/321, 3.43%), *DUOX2* (10/321, 3.12%), *PAH* (8/321, 2.49%) and SLC26A4 (8/321, 2.49%) were the top five genes with the highest carrier frequencies of P/LP mutations (Supplementary Table 8). Five newborns were detected with a loss of one copy of *SMN1* using a customized copy number caller.

For the 164 primary immunodeficiency disorders recognized by the IUIS, 9 heterozygous P/LP variants in 6 genes, corresponding to 6 diseases, were identified in 9 of the 321 newborn children (2.80%), all in a heterozygous state (Table 2). Of these, there are two genes (*ADA* and *JAK3*) for which we found two carriers individually. In contrast, no P/LP mutations were detected in *IL-2RG*, which was previously reported as the most common PID gene by two Southern China SCID cohort studies ^20^, but our sample size was relatively small. Notably, we also identified a likely pathogenic variant in *TNFRSF13B* (NM_012452.2, c.542C>A, p.A181E) in two children, and the variant was reported to be associated with Primary Immunodeficiency (specifically Common Variable Immunodeficiency) with incomplete penetrance. This mutation was included in the final report of infants and follow-ups will be conducted until three years of age.

In addition to P/LP variants, variants of uncertain significance were also identified and reviewed following the standards and guidelines of ACMG/AMP. The heterozygous null variant in the gene *SLC25A13* (c.2T>C, p.M1T) was identified in four children, which was predicted as start loss and likely affecting the initiator methionine of the SLC25A13 mRNA which might result in Type II Citrullinemia ^21,22^.Two newborns were detected carrying a VUSs of c.-4C>T, - in ASS1, likely to be associated with Type I Citrullinemia ^23,24^(Supplementary Table 9). Although these VUSs were not included in the final report for the participants, follow-up of children with VUSs will be conducted as a part of routine follow-up for participants until three years of age.

Sanger sequencing was conducted to confirm P/LP and VUS variants identified by newborn WGS. In general, 143 out of 145 mutations were validated correctly, therefore the precision of newborn WGS on selected inherited disorders was 98.62%. Carriers of one copy loss of gene *SMN1* were validated by MLPA and qPCR, showing that five out of six predicted carriers were true. The results of validation were summarized in Supplementary Table 10.

## ***Actionable PGx profiling***

A total of 730 actionable PGx variants associated with 5 essential pharmacogenes were detected by newborn WGS (Table 3). We observed that 313 newborns (97.51%) carried at least one actionable PGx variant, while 193 newborns (60.12%) harbored two and/or three variants (Fig. 1C). Unsurprisingly, the *CYP2D6* gene had the highest carrier frequency, where 266 out of 321 infants (82.87%) harbored at least one actionable PGx variant. The gene *CYP2C19* showed the second-highest carrier rate, where 209 infants (65.11%) carried at least one clinically relevant variant. In addition, 133 and 122 infants carried actionable PGx variants at the gene *UGT1A1* and *NUDT15,* respectively. No actionable variant was identified in the *DPYD* gene in the 321 children.

We further compared the allele frequency of actionable PGx variants between the Qingdao cohort and five subpopulations of the 1000 Genome dataset, including East Asians (EAS), South Asians (SAS), Africans (AFR), Europeans (EUR), and Americans (AMR) (Fig.2B and Supplementary Table 11). In most cases, the allele frequency of the Qingdao cohort is consistent with the EAS, but differed significantly with the SAS, the AFR, the EUR and the AMR, such as *CYP2C19*2, CYP2C19*3* and *NUDT15*6*. It should be noted, however, that three common PGx variants in the Qingdao cohort, *CYP2D6*10* (48.60%), *NUDT15*3 (*13.08%) and *UGT1A1*6* (21.18%), showed significant frequency differences with the EAS (P<0.05), indicating population diversity within the East Asians. Notably, two rare variants, *CYP2D6*8* (1.87%, n=11) and *CYP2C19*6* (0.16%, n=1) which have not been reported in any subpopulation in the 1000 Genome phase 3 dataset and were firstly detected in our Qingdao 321 newborns.

Sanger sequencing was performed in a number of randomly selected actionable PGx variants (235 out of 730, 30%) detected by newborn WGS. In total, 234 variants were validated to be true, whereas one homozygous variant in the *CYP2D6 *41* was found to be heterozygous (Supplementary Table 10). Therefore, the precision of newborn WGS in PGx profiling was 99.57%.

# Supplementary Discussion

In the present study, 321 non-pre-selected newborns from three hospitals of Qingdao in China were genetically screened by newborn WGS, resulting in the identification of 131 P/LP variants and 5 pathogenic CNVs in 59 inherited metabolic diseases, 9 heterozygous P/LP variants in 164 PID´s and 730 actionable PGx variants.

Conducting newborn screening using genomic approaches has been controversial due to the technical concerns of choosing genes/diseases to be investigated and interpreting results, as well as ethical issues such as violating personal privacy and changing the public health core ^25^. Thus, the ACMG guideline of clinical application of genomic sequencing published in 2012 did not endorse the use of exome sequencing or genomic sequencing as a first line method for newborn screening ^26^. However, the current RUSP for newborn screening suffers from a slow, condition-by-condition review process, which is more and more challenged by patient advocates and rapidly improving screening technologies^27^. In recent years, research-based newborn expanded screening has been published to explore the feasibility and benefits of future NBS. For instance, the BabySeq project was set up to compare the standard newborn healthcare with genomic sequencing, and a curated list of 954 genes was suggested to be suitable for newborn testing reports, including 884 genes with strong/definitive evidence for highly penetrant childhood-onset diseases and 70 additional genes with moderate penetrance associated conditions that actionable in the childhood as well as genes with strong pharmacogenomic associations ^28^. Previous studies using WES/WGS on newborns have used an indiscriminate inclusion of OMIM defined diseases ^7,8^. In our study, however, we selected a limited set of disorders containing the currently recommended 59 inherited disorders, 164 selected primary immunodeficiency disorders and five genes associated with adverse drug reactions, thus limiting the search for genetic variants with known clinical importance. As expected, most disorders examined in our study were also included in the category A of the Babyseq curated list, though a few disease-gene pairs suggested by the RUSP were not recommended by the Babyseq list due to moderate penetrance in childhood, such as short-chain acyl-CoA dehydrogenase deficiency and the causative gene *ACADS* as well as hearing loss and the genes *GJB3 and MT-RNR1*.

Expanded newborn screening by MS/MS as a non-mandatory complement of routine NBS tests in mainland China is currently conducted in a few regions, such as Shanghai, Guangdong and Zhejiang ^29,30^. The overall incidence of inborn errors metabolism (IEM) detected by MS/MS were estimated to be 38.69 per 100,000 births, though the number might be underestimated due to that the panel varies between centers ^31^. By contrast, the newborn WGS in the current study following the recommended uniform screening panel which covered 59 inherited disorders corresponding to an accumulative incidence of 225 in 100,000 births (see Supplementary Table 2), suggesting a major improvement in detectability ^31-37^. Additionally, combing with the alternative CNV pipeline, WGS identified five carriers of SMA in 321 newborns, indicating the potential of WGS in common pathogenic CNVs screening in the neonatal population. According to previous studies, the accumulative prevalence of SCIDs worldwide was around 1 in 58,000 - 100,000 live births. However, given the lack of routine neonatal SCIDs testing, it is difficult to accurately estimate their incidence in China. Children with SCID are often not detected until they have symptoms of severe infection. Advances in technologies like WGS, could facilitate early detection and better care of PIDs in the mainland of China.

Notably, all the positive findings of routine NBS were recalled correctly without missing any variant. Moreover, the child with a false positive C5OH result was confirmed to be a carrier of 3-Methylcrotonyl-CoA Carboxylase Deficiency by WGS. It should be noted that newborn WGS additionally detected two infants carrying compound heterozygous variants in *GJB2* and four children with homoplasmy pathogenic mutations in *MT-RNR1*, indicating an increased risk of late-onset deafness or drug-induced hearing loss, respectively. Previous studies reorted that homozygous or compound heterozygous variants of c.109G>A were associated with mild to moderate deafness, and showed incomplete penetrance, which can lead to late-onset deafness ^38,39^. Meanwhile, homoplasmic m.1095T>C mutation in MT-RNR1 was associated with a risk of aminoglycoside-induced hearing loss ^40^. The results of newborn WGS suggested that genomic sequencing significantly improves the detection rate of disease-causing variants as well as VUSs which was helpful for further follow-up and early intervention. Early identification of high-risk children and carriers has greatly increased the diagnostic yield and altered their clinical management, thus providing benefits of newborn WGS for research participants and their families.

One important aspect when screening for disorders in a given population is to use a matched control database as variants can be highly specific for a given ethnic group ^41,42^. Most databases published to date are based on individuals of European descent and many populations are limited, or every poorly represented. The Genome Asia 100K project ^43^ aims to address this gap by sequencing a large number of individuals from different Asian populations and can be used as a reference and is essential as an unexpectedly high allele frequency of a given variant may be restricted to a particular ethnic population. In China, the incidence of inherited disease differs greatly among regions. Investigating the local disease-associated mutations spectrum will be useful for planning future NBS programs. Previous MS/MS studies revealed that MMA, PKU and primary carnitine deficiency were ranked as the top three common IMEs in the city of Jining in the Shandong province ^44^. In our 321 newborns from Qingdao, also located in the Shandong province, the common diseases were as follows (in order): hearing loss, methylmalonic acidemia, congenital hypothyroidism, PKU, carnitine defect, spinal muscular atrophy, congenital adrenal hyperplasia and tetrahydrobiopterin deficiencies (Supplementary Table 8). Albeit the most common diseases seemed consistent with the Jining study, WGS generated a much more comprehensive disease-mutation spectrum in a wide range of inherited diseases.

Interestingly, 97.51% assessed child harbored at least one actionable PGx variant, suggesting a major potential for improvement of personalized drug safety for children. This result was in line with one European 44,000 biobank participants study, where “99.8% of the participants had a genotype associated with increased risks to at least one medication” ^45^. Moreover, a retrospective analysis of the 1000 Genome dataset on 120 pharmacogenomics genes across 26 global populations have reported a median of three clinical variants per individual, and East Asian showed the highest percentage of loss-of-function variants (60.9%) ^46^. Among the selected gene-drug pairs, irinotecan, azathioprine, mercaptopurine and tioguanine were prescription drugs to pediatric patients, while codeine and clopidogrel are restricted for use of children under 18 years old ^47-49^. Emerging studies have shown an increased risk of severe toxicity and adverse drug reactions in pediatric patients with the *UGTA1*6* genotype when receiving irinotecan-based chemotherapy and children with acute lymphoblastic leukemia carrying *NUDT15*3* mutation receiving azathioprine, mercaptopurine or tioguanine ^48,49^. In our Qingdao cohort, the allele frequency of *UGT1A1**6 and *NUDT15*3* was 21.18% and 13.08% respectively, which is also consistent with previous findings that these mutations were common in east Asians although rare in Europeans (Table 3 and Fig. 2B). Furthermore, clopidogrel is broadly utilized primarily for the management of coronary artery and peripheral vascular disease, corresponding to approximately 5-8% and 10-20% worldwide prevalence^50^, respectively. In the current study, we observed that nearly 62.31% of the children were carriers of loss-of-function variants (*2 or *3) at the *CYP2C19* gene, indicating a risk of decreased efficiency when receiving clopidogrel treatment as adults. In addition, we identified 81 infants with *CYP2D6**10 in the homozygous state, suggesting nearly 25% of participants were poor metabolizers in drug metabolism, including codeine ^51,52^. Although PGx profiling was included in the BabySeq project in the United States, no neonatal study has been conducted in China. Due to the lifelong use of pharmacogenomics data, our findings suggest that participants may obtain benefits from PGx profiling already in early childhood.

Limitations of the current study are the small sample size and restricted metabolic tests which might lead to an incomplete view of clinical sensitivity of WGS as well as carrier frequency in newborns. A large-scale NBS effort is needed to validate our findings in the current study and fully investigate the treatable or curable medical conditions in newborns. The technical challenge of newborn WGS is to screen genes with high homology due to the misalignment of short-read sequencing. Therefore, the customized pipeline is needed to improve the accuracy and sensitivity of SNVs at genes with high-level homology. Albeit the present seqeucing cost and turnaround time of WGS is several times compared to the present NBS methods, it is foreseen that the pitfalls will improve with time which will facilitate the application of newborn WGS in NBS programs. Our work suggested that selective identification of genomic data, where therapeutic options are available, does not violate the Wilson-Jungner criteria^53,54^ and also provides a basis for future research on variants in an expanding number of genes and should therefore be considered in future screening programs for all newborns. Further discussion of the accuracy of interpretation and ethical use of genmic information needs to take place on a global scale.

# Reference

1. Folling A, Sydnes S. A method for the estimation of phenylpyruvic acid in urine with some examples of its use in dietary treatment of phenylpyruvic oligophrenia. *Scand J Clin Lab Invest*. 1958;10(4):355-8. doi:10.3109/00365515809051236

2. Scriver CC. A simple phenylalanine method for detecting phenylketonuria in large populations of newborn infants, by Robert Guthrie and Ada Susi, Pediatrics, 1963;32:318-343. *Pediatrics*. Jul 1998;102(1 Pt 2):236-7.

3. Wilson JMG, Jungner G, World Health O. Principles and practice of screening for disease / J. M. G. Wilson, G. Jungner. Geneva: World Health Organization; 1968.

4. Chan K, Puck JM. Development of population-based newborn screening for severe combined immunodeficiency. *J Allergy Clin Immunol*. Feb 2005;115(2):391-8. doi:10.1016/j.jaci.2004.10.012

5. Amatuni GS, Currier RJ, Church JA, et al. Newborn Screening for Severe Combined Immunodeficiency and T-cell Lymphopenia in California, 2010-2017. *Pediatrics*. Feb 2019;143(2)doi:10.1542/peds.2018-2300

6. Nakagawa N, Imai K, Kanegane H, et al. Quantification of κ-deleting recombination excision circles in Guthrie cards for the identification of early B-cell maturation defects. *J Allergy Clin Immunol*. Jul 2011;128(1):223-225.e2. doi:10.1016/j.jaci.2011.01.052

7. Ceyhan-Birsoy O, Murry JB, Machini K, et al. Interpretation of Genomic Sequencing Results in Healthy and Ill Newborns: Results from the BabySeq Project. *Am J Hum Genet*. Jan 3 2019;104(1):76-93. doi:10.1016/j.ajhg.2018.11.016

8. Pavey AR, Bodian DL, Vilboux T, et al. Utilization of genomic sequencing for population screening of immunodeficiencies in the newborn. *Genet Med*. Dec 2017;19(12):1367-1375. doi:10.1038/gim.2017.57

9. Dai L, Zhu J, Liang J, Wang YP, Wang H, Mao M. Birth defects surveillance in China. *World J Pediatr*. Nov 2011;7(4):302-10. doi:10.1007/s12519-011-0326-0

10. Mei L, Song P, Xu L. Newborn screening and related policy against Phenylketonuria in China. *Intractable Rare Dis Res*. Aug 2013;2(3):72-6. doi:10.5582/irdr.2013.v2.3.72

11. Li Z, Huang L, Du C, et al. Analysis of the Screening Results for Congenital Adrenal Hyperplasia Involving 7.85 Million Newborns in China: A Systematic Review and Meta-Analysis. Systematic Review. *Frontiers in Endocrinology*. 2021-April-23 2021;12(365)doi:10.3389/fendo.2021.624507

12. Wang Q, Xiang J, Sun J, et al. Nationwide population genetic screening improves outcomes of newborn screening for hearing loss in China. *Genetics in Medicine*. 2019/10/01 2019;21(10):2231-2238. doi:10.1038/s41436-019-0481-6

13. Xu Y, Lin Z, Tang C, et al. A new massively parallel nanoball sequencing platform for whole exome research. *BMC bioinformatics*. Mar 25 2019;20(1):153. doi:10.1186/s12859-019-2751-3

14. Drmanac R, Sparks AB, Callow MJ, et al. Human genome sequencing using unchained base reads on self-assembling DNA nanoarrays. *Science*. Jan 1 2010;327(5961):78-81. doi:10.1126/science.1181498

15. Huang J, Liang X, Xuan Y, et al. A reference human genome dataset of the BGISEQ-500 sequencer. *GigaScience*. May 1 2017;6(5):1-9. doi:10.1093/gigascience/gix024

16. Picard C, Bobby Gaspar H, Al-Herz W, et al. International Union of Immunological Societies: 2017 Primary Immunodeficiency Diseases Committee Report on Inborn Errors of Immunity. *J Clin Immunol*. Jan 2018;38(1):96-128. doi:10.1007/s10875-017-0464-9

17. Bousfiha A, Jeddane L, Picard C, et al. The 2017 IUIS Phenotypic Classification for Primary Immunodeficiencies. *J Clin Immunol*. Jan 2018;38(1):129-143. doi:10.1007/s10875-017-0465-8

18. Chen X, Sanchis-Juan A, French CE, et al. Spinal muscular atrophy diagnosis and carrier screening from genome sequencing data. *Genetics in Medicine*. 2020/05/01 2020;22(5):945-953. doi:10.1038/s41436-020-0754-0

19. Richards S, Aziz N, Bale S, et al. Standards and guidelines for the interpretation of sequence variants: a joint consensus recommendation of the American College of Medical Genetics and Genomics and the Association for Molecular Pathology. *Genet Med*. May 2015;17(5):405-24. doi:10.1038/gim.2015.30

20. Lee PP, Chan KW, Chen TX, et al. Molecular diagnosis of severe combined immunodeficiency--identification of IL2RG, JAK3, IL7R, DCLRE1C, RAG1, and RAG2 mutations in a cohort of Chinese and Southeast Asian children. *Journal of clinical immunology*. Apr 2011;31(2):281-96. doi:10.1007/s10875-010-9489-z

21. Zeng HS, Zhao ST, Deng M, et al. Inspissated bile syndrome in an infant with citrin deficiency and congenital anomalies of the biliary tract and esophagus: identification and pathogenicity analysis of a novel SLC25A13 mutation with incomplete penetrance. *Int J Mol Med*. Nov 2014;34(5):1241-8. doi:10.3892/ijmm.2014.1929

22. Lin WX, Zeng HS, Zhang ZH, et al. Molecular diagnosis of pediatric patients with citrin deficiency in China: SLC25A13 mutation spectrum and the geographic distribution. *Sci Rep*. Jul 11 2016;6:29732. doi:10.1038/srep29732

23. Engel K, Höhne W, Häberle J. Mutations and polymorphisms in the human argininosuccinate synthetase (ASS1) gene. *Hum Mutat*. Mar 2009;30(3):300-7. doi:10.1002/humu.20847

24. Diez-Fernandez C, Rüfenacht V, Häberle J. Mutations in the Human Argininosuccinate Synthetase (ASS1) Gene, Impact on Patients, Common Changes, and Structural Considerations. *Hum Mutat*. May 2017;38(5):471-484. doi:10.1002/humu.23184

25. Goldenberg AJ, Sharp RR. The ethical hazards and programmatic challenges of genomic newborn screening. *Jama*. Feb 1 2012;307(5):461-2. doi:10.1001/jama.2012.68

26. Points to consider in the clinical application of genomic sequencing. *Genet Med*. Aug 2012;14(8):759-61. doi:10.1038/gim.2012.74

27. Bailey DB, Jr., Gehtland L. Newborn screening: evolving challenges in an era of rapid discovery. *Jama*. Apr 21 2015;313(15):1511-2. doi:10.1001/jama.2014.17488

28. Ceyhan-Birsoy O, Machini K, Lebo MS, et al. A curated gene list for reporting results of newborn genomic sequencing. *Genet Med*. Jul 2017;19(7):809-818. doi:10.1038/gim.2016.193

29. Huang X, Yang L, Tong F, Yang R, Zhao Z. Screening for inborn errors of metabolism in high-risk children: a 3-year pilot study in Zhejiang Province, China. *BMC Pediatr*. Feb 24 2012;12:18. doi:10.1186/1471-2431-12-18

30. Gu X, Wang Z, Ye J, Han L, Qiu W. Newborn screening in China: phenylketonuria, congenital hypothyroidism and expanded screening. *Ann Acad Med Singap*. Dec 2008;37(12 Suppl):107-4.

31. Deng K, Zhu J, Yu E, et al. Incidence of inborn errors of metabolism detected by tandem mass spectrometry in China: A census of over seven million newborns between 2016 and 2017. *J Med Screen*. Sep 2021;28(3):223-229. doi:10.1177/0969141320973690

32. Feuchtbaum L, Carter J, Dowray S, Currier RJ, Lorey F. Birth prevalence of disorders detectable through newborn screening by race/ethnicity. *Genet Med*. Nov 2012;14(11):937-45. doi:10.1038/gim.2012.76

33. He J, Tang M, Zhang X, et al. Incidence and prevalence of 121 rare diseases in China: Current status and challenges. *Intractable Rare Dis Res*. May 2019;8(2):89-97. doi:10.5582/irdr.2019.01066

34. Chien YH, Chiang SC, Chang KL, et al. Incidence of severe combined immunodeficiency through newborn screening in a Chinese population. *J Formos Med Assoc*. Jan 2015;114(1):12-6. doi:10.1016/j.jfma.2012.10.020

35. Verhaart IEC, Robertson A, Wilson IJ, et al. Prevalence, incidence and carrier frequency of 5q-linked spinal muscular atrophy - a literature review. *Orphanet J Rare Dis*. Jul 4 2017;12(1):124. doi:10.1186/s13023-017-0671-8

36. Huang YL, Sheng HY, Jia XF, et al. [GAA gene variants and genotype-phenotype correlations in patients with glycogen storage disease type Ⅱ]. *Zhonghua Er Ke Za Zhi*. Mar 2 2021;59(3):189-194. doi:10.3760/cma.j.cn112140-20200710-00710

37. Zhou Y, Li C, Li M, et al. Mutation analysis of common deafness genes among 1,201 patients with non-syndromic hearing loss in Shanxi Province. *Mol Genet Genomic Med*. Mar 2019;7(3):e537. doi:10.1002/mgg3.537

38. Shen N, Peng J, Wang X, et al. Association between the p.V37I variant of GJB2 and hearing loss: a pedigree and meta-analysis. *Oncotarget*. 2017;8(28):46681-46690. doi:10.18632/oncotarget.17325

39. Cai L, Liu Y, Xu Y, et al. Multi-Center in-Depth Screening of Neonatal Deafness Genes: Zhejiang, China. *Frontiers in genetics*. 2021;12:637096-637096. doi:10.3389/fgene.2021.637096

40. Barbarino JM, McGregor TL, Altman RB, Klein TE. PharmGKB summary: very important pharmacogene information for MT-RNR1. *Pharmacogenet Genomics*. Dec 2016;26(12):558-567. doi:10.1097/fpc.0000000000000247

41. Auton A, Brooks LD, Durbin RM, et al. A global reference for human genetic variation. *Nature*. Oct 1 2015;526(7571):68-74. doi:10.1038/nature15393

42. Whiffin N, Ware JS, O'Donnell-Luria A. Improving the understanding of genetic variants in rare disease with large-scale reference populations. *JAMA: Journal of the American Medical Association*. // 2019;322:1305-1306. doi:10.1001/jama.2019.12891

43. GenomeAsia KC. The GenomeAsia 100K Project enables genetic discoveries across Asia. *Nature*. Dec 2019;576(7785):106-111. doi:10.1038/s41586-019-1793-z

44. Guo K, Zhou X, Chen X, Wu Y, Liu C, Kong Q. Expanded Newborn Screening for Inborn Errors of Metabolism and Genetic Characteristics in a Chinese Population. *Front Genet*. 2018;9:122. doi:10.3389/fgene.2018.00122

45. Reisberg S, Krebs K, Lepamets M, et al. Translating genotype data of 44,000 biobank participants into clinical pharmacogenetic recommendations: challenges and solutions. *Genetics in Medicine*. 2019/06/01 2019;21(6):1345-1354. doi:10.1038/s41436-018-0337-5

46. Wright GEB, Carleton B, Hayden MR, Ross CJD. The global spectrum of protein-coding pharmacogenomic diversity. *The Pharmacogenomics Journal*. 2018/01/01 2018;18(1):187-195. doi:10.1038/tpj.2016.77

47. Tobias JD, Green TP, Coté CJ. Codeine: Time to Say "No". *Pediatrics*. Oct 2016;138(4)doi:10.1542/peds.2016-2396

48. Pereira NL, Rihal CS, So DYF, et al. Clopidogrel Pharmacogenetics. *Circ Cardiovasc Interv*. 2019;12(4):e007811-e007811. doi:10.1161/CIRCINTERVENTIONS.119.007811

49. Zhu Y, Yin D, Su Y, et al. Combination of common and novel rare NUDT15 variants improves predictive sensitivity of thiopurine-induced leukopenia in children with acute lymphoblastic leukemia. *Haematologica*. 2018;103(7):e293-e295. doi:10.3324/haematol.2018.187658

50. Bauersachs R, Zeymer U, Brière JB, Marre C, Bowrin K, Huelsebeck M. Burden of Coronary Artery Disease and Peripheral Artery Disease: A Literature Review. *Cardiovasc Ther*. 2019;2019:8295054. doi:10.1155/2019/8295054

51. Crews KR, Gaedigk A, Dunnenberger HM, et al. Clinical Pharmacogenetics Implementation Consortium (CPIC) guidelines for codeine therapy in the context of cytochrome P450 2D6 (CYP2D6) genotype. *Clin Pharmacol Ther*. Feb 2012;91(2):321-6. doi:10.1038/clpt.2011.287

52. Ingelman-Sundberg M, Sim SC, Gomez A, Rodriguez-Antona C. Influence of cytochrome P450 polymorphisms on drug therapies: pharmacogenetic, pharmacoepigenetic and clinical aspects. *Pharmacol Ther*. Dec 2007;116(3):496-526. doi:10.1016/j.pharmthera.2007.09.004

53. Wilson JM, Jungner YG. [Principles and practice of mass screening for disease]. *Bol Oficina Sanit Panam*. Oct 1968;65(4):281-393. Principios y metodos del examen colectivo para identificar enfermedades.

54. Petros M. Revisiting the Wilson-Jungner criteria: how can supplemental criteria guide public health in the era of genetic screening? *Genet Med*. Jan 2012;14(1):129-34. doi:10.1038/gim.0b013e31823331d0
